# Comparative transcriptomics reveals genes commonly induced by distinct stressors in *Chlamydia*

**DOI:** 10.1128/iai.00758-25

**Published:** 2026-02-20

**Authors:** Ronald Haines, Danny Wan, Guangming Zhong, Huizhou Fan

**Affiliations:** 1 Department of Pharmacology, Robert Wood Johnson Medical School, Rutgers, The State University of New Jersey, Piscataway, New Jersey, USA; 2 Department of Microbiology and Immunology, University of Texas Health San Antonio 14742 , San Antonio, Texas, USA; University of California Davis, Davis, California, USA

**Keywords:** *Chlamydia*, stress response, transcriptomics, comparative transcriptomics, RNA-Seq, heat shock, interferon-γ, iron starvation, tryptophan starvation, β-lactam

## Abstract

*Chlamydia trachomatis* is a leading cause of urogenital infections that can result in serious long-term complications. This obligate intracellular bacterium undergoes a biphasic developmental cycle alternating between the infectious elementary body and the replicative reticulate body and can enter a persistent state in response to adverse environmental conditions. Although transcriptomic reprogramming is central to chlamydial stress adaptation and persistence, how responses differ across biologically distinct stressors remains incompletely defined. Here, we performed a comparative reanalysis of five published, high-quality *C. trachomatis* RNA-Seq data sets generated under prolonged interferon-γ treatment, tryptophan starvation, iron starvation, penicillin exposure, or acute heat shock. Global transcriptomic analyses reveal stress-specific reprogramming and a clear separation between the transcriptome induced by heat shock and those induced by chronic stresses. Transcriptomic overlap observed among chronic stress conditions is substantially reduced when the heat shock transcriptome is included, indicating that shared transcriptional features are stressor-dependent. Consistent with prior findings, tryptophan starvation and iron starvation exhibit particularly close transcriptomic similarity, likely reflecting regulatory cross-talk mediated by the iron-dependent transcriptional repressor YtgR. Notably, this similarity exceeds that observed between tryptophan starvation and interferon-γ treatment, despite the well-established role of interferon-γ in inducing host-mediated tryptophan depletion. In contrast, interferon-γ induces a distinct but partially overlapping transcriptome, likely reflecting activation of additional host-mediated antimicrobial mechanisms beyond tryptophan deprivation. Together, these findings demonstrate that adaptation to different biological stressors in *C. trachomatis* is driven by distinct transcriptomic reprogramming, while consistently involving a subset of functions that may represent points of vulnerability for disrupting chlamydial persistence.

## INTRODUCTION

Bacteria have evolved strategies to respond to environmental challenges, including nutrient deprivation, sudden temperature fluctuations, and exposure to antibiotics. These adaptive responses are critical for bacteria to survive and thrive in hostile environments (1–3). Pathogenic bacteria, in particular, face additional pressures from host immune defenses and often deploy mechanisms to evade these responses (3). Understanding how bacteria reprogram their cellular functions in response to stress is crucial for deciphering their survival strategies and may aid in the development of novel therapies.

*Chlamydia trachomatis* is an obligate intracellular bacterial pathogen known for causing pelvic inflammatory disease, infertility, ectopic pregnancy, and abortion in women (4–6). Increasing evidence also implicates *C. trachomatis* infection as a contributor to male infertility (7, 8). In addition, ocular serovars of *C. trachomatis* cause trachoma, a major cause of preventable blindness in resource-limited regions worldwide.

*Chlamydia* has a developmental cycle that alternates between the infectious elementary body (EB) and the proliferative reticulate body (RB) (4, 6, 9–11). EBs enter host cells and differentiate into RBs inside cytoplasmic vacuoles known as inclusions. Following multiple rounds of replication, RBs differentiate back into EBs, which exit host cells through either cell rupture or inclusion extrusion (4, 6, 9–11).

Adverse environmental conditions can disrupt this developmental cycle and induce a persistent state characterized by the formation of enlarged, non-dividing RBs, often referred to as aberrant bodies (12–21). During persistence, RB replication and EB production are suppressed, allowing chlamydiae to survive prolonged stress. Importantly, persistence is reversible. Upon stress removal, aberrant bodies revert to RBs, which resume replication and re-enter the productive developmental cycle. Persistence is a major cause of clinical treatment failure and chronic chlamydial infections, contributing to infertility and other complications (12–21).

Chlamydial persistence has been modeled in cell culture using a variety of stressors relevant to infection and therapy, including interferon-γ (IFNγ), β-lactam antibiotics, nutrient deprivation, iron starvation, and heat shock (12, 14–19, 22–25). IFNγ, produced by activated immune cells, inhibits RB replication and EB formation and is a well-established persistence-inducing signal *in vitro* and *in vivo*, reflecting its central role in host-mediated antimicrobial defense (24–26). β-Lactam antibiotics such as amoxicillin, which may be administered during pregnancy or co-infection, are potent inducers of persistence despite not being a first-line antichlamydial agent (15–18, 24). Conditions that limit nutrient availability, including iron limitation, can restrict chlamydial growth and development (14, 25, 27). Febrile responses are more characteristic of infections caused by *C. trachomatis* lymphogranuloma venereum serovars or the zoonotic pathogen *Chlamydia psittaci* (28–32) and may expose bacteria to repeated episodes of elevated temperatures that disrupt protein homeostasis (22, 33, 34).

Stress responses in *C. trachomatis* are governed by transcriptomic reprogramming (12, 14, 17, 22, 25). Previous studies have shown overlaps in transcriptomic changes induced by specific stressors, such as iron starvation and tryptophan depletion (25), or β-lactam antibiotics and IFNγ (17). However, how transcriptional responses compare across a broader range of chronic and acute stress conditions, and the extent to which similarities or differences reflect shared or distinct challenges, remains incompletely defined (22).

To address this gap, we performed a comparative reanalysis of published *C. trachomatis* RNA-Seq data sets generated under five stress conditions: IFNγ treatment (17), iron starvation (25), tryptophan starvation (25), penicillin exposure (17), and heat shock (22). By systematically comparing these transcriptomes using a unified analytical approach, this study demonstrates that adaptation of *C. trachomatis* to diverse biological stressors is driven by distinct transcriptional programs. It further establishes that a set of regulated genes is consistently involved across stress conditions and may represent shared vulnerabilities that may be exploited for therapeutic intervention.

## RESULTS

### Exceptionally high sequencing depths of stress transcriptomic studies

We analyzed RNA-Seq data sets from *C. trachomatis* cultures exposed to five distinct stress conditions: the antichlamydial cytokine IFNγ, the β-lactam antibiotic penicillin, iron starvation induced by the chelator 2,2-bipyridyl, tryptophan starvation using a tryptophan-free medium, and heat shock (17, 22, 25). The IFNγ and penicillin stress RNA-Seq data sets were published by the laboratory of Jan Rupp (17); the iron starvation and tryptophan starvation data sets by the laboratory of Ray A. Carabeo (25); and the heat shock data set by the Fan Lab (22). Key experimental parameters are summarized in Table 1. The IFNγ and penicillin stress studies utilized *C. trachomatis* serovar D (17), whereas the remaining studies employed serovar L2 (22, 25). Human cervical carcinoma HeLa cells served as the host cell line in all experiments except for the heat shock study, which used mouse fibroblast L929 cells (17, 22, 25).

**TABLE 1:** Experimental conditions, data set sources, and depths for the stress transcriptomic studies analyzed^*a*^

Study	Ct organism	Host cell	Treatment and duration	Raw RNA-Seq data accession no.	Mapped Ct reads	Depth (× coverage)
Interferon-γ (17)	Serovar D (UW-3/Cx)	HeLa	Control medium	SRR5834397, SRR5834399	16,800,722 9,755,404	1,600 929
			50 U/mL IFN-γ, −24 to 24 hpi	SRR13189638, SRR13189738	2,557,223 4,331,636	244 413
Penicillin (17)	Serovar D UW-3/Cx	HeLa	*Control medium*	SRR5834397, SRR5834399	16,800,722 9,755,404	1,600 929
			1 U/mL penicillin, 0 to 24 hpi	SRR5834398, SRR5834400	31,724,401 18,660,673	3,021 1,777
Iron starvation (25)	Serovar L2 (434/BU)	HeLa	Control medium	GSE179003	118,754,751	8,512
			100 µM BPD, 0 to 24 hpi	GSE179003	42,374,135	3,037
Tryptophan starvation (25)	Serovar L2 (434/BU)	HeLa	Control medium	GSE179003	118,754,751	8,512
			Trp-free medium, 0 to 24 hpi	GSE179003	16,509,535	1,183
Heat shock (22)	Serovar L2 (434/BU)	L929	37°C	GSE173366	5,435,469	260
			45°C, 15.5 to 16 hpi	GSE173366	5,681,297	271

^*a*^ For IFNγ and penicillin treatments, duplicate biological samples were sequenced separately and deposited under distinct accession numbers. For iron and tryptophan starvation studies, triplicate biological samples were sequenced and deposited under a single accession number. Abbreviations: Ct, *C. trachomatis*; hpi, hours postinoculation; BPD, 2,2-bipyridyl.

Tryptophan starvation, iron starvation, and penicillin treatment were applied after inoculation, whereas IFNγ treatment of host cells was initiated 24 h prior to inoculation. Tryptophan- or iron-starved and IFNγ- or penicillin-treated cultures were harvested at 24 h postinoculation for RNA extraction. Heat shock treatment at 45°C was conducted between 15.5 and 16 h postinoculation (22). Based on the length of stress exposure, we characterize the first four transcriptomes as chronic stress transcriptomes and heat shock as an acute stress transcriptome.

To ensure comparability across studies, we reprocessed all raw sequencing data using a consistent bioinformatic workflow and identical software tools, rather than relying on previously published secondary analyses. All five RNA-Seq data sets achieved extremely high sequencing depths (Table 1), with genome coverage ranging from nearly 250-fold to over 8,000-fold. These high coverages support rigorous comparisons of *Chlamydia* transcriptomic responses across diverse stress conditions.

### Extensive transcriptomic reprogramming in stress responses

The *C. trachomatis* genome encodes more than 900 protein-coding genes. DESeq analysis revealed that exposure to IFNγ, tryptophan starvation, iron starvation, penicillin, or heat shock markedly altered the expression of nearly one-third of these genes, ranging from 292 to 363 differentially expressed genes (DEGs) at a ≥1.5-fold change and *P* < 0.05 threshold (a cutoff commonly used in bacterial transcriptomic studies and employed in the original analyses of these data sets) (Table 2). These findings reinforce previous observations that *C. trachomatis* mounts extensive transcriptomic reprogramming when confronted with diverse forms of stress (12, 14, 17, 22, 25).

**TABLE 2:** Numbers of upregulated and downregulated genes within each stress transcriptome

Stressor	Upregulated genes	Downregulated genes	All DEGs
Interferon-γ	163	200	363
Iron starvation	136	181	317
Tryptophan starvation	154	189	343
Penicillin	92	200	292
Heat shock	171	156	325

### Distinction of IFNγ, tryptophan starvation, iron starvation, or penicillin stress transcriptomes from the heat shock transcriptome

**Fig 1:**
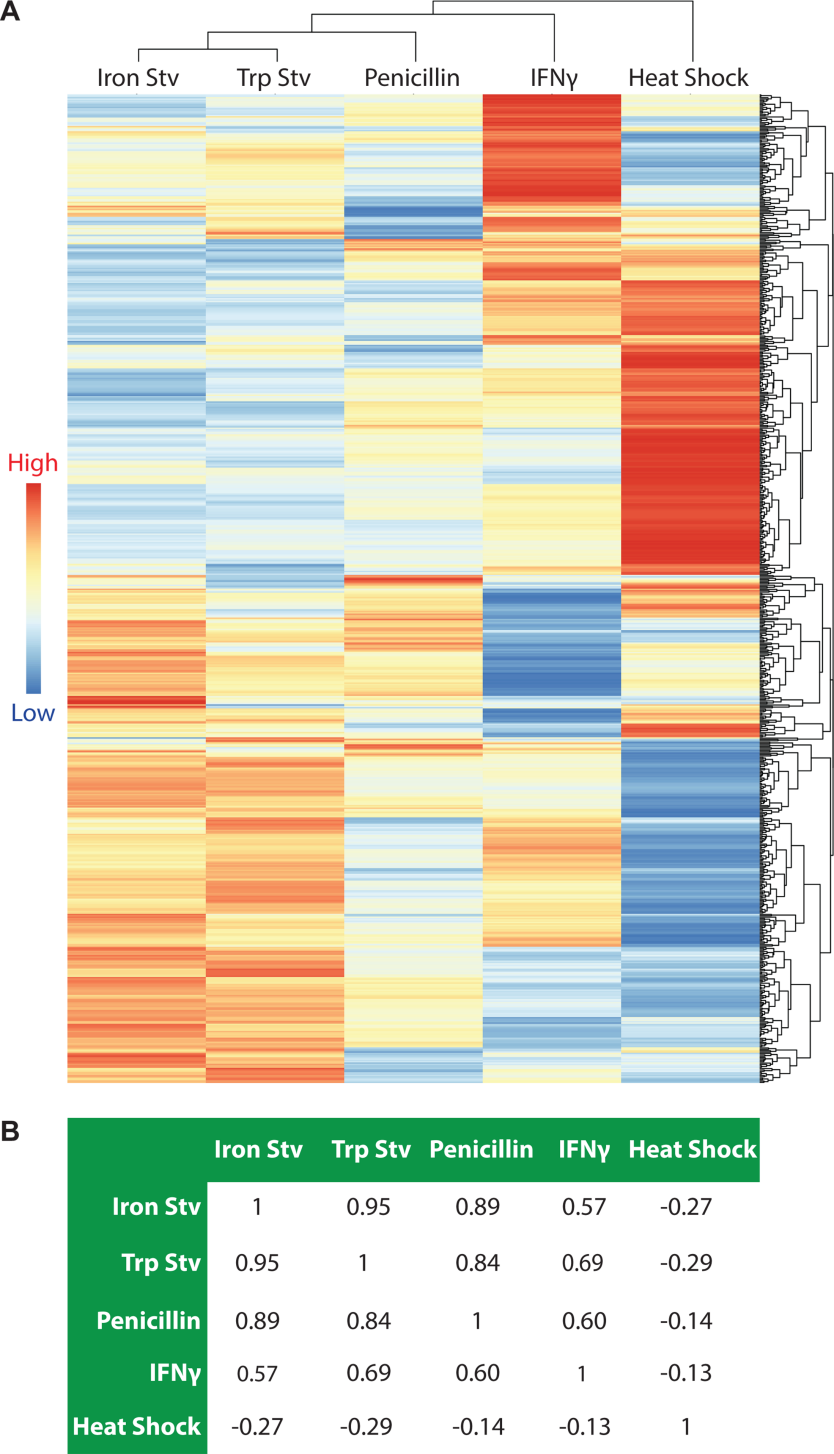
Acute heat shock and chronic stressors elicit distinct transcriptomic responses in *C. trachomatis*. (**A**) Hierarchical clustering heatmap of 667 genes differentially expressed in at least one stress transcriptome (adjusted *P* < 0.05; |log_2_ fold change| ≥ 0.585). Each row represents a gene, and each column represents a stressor. The dendrograms depict the relative similarity among genes (rows) and among stress transcriptomes (columns) based on their overall expression patterns across conditions. Heatmap colors represent log₂ fold change values relative to the corresponding control condition. The heatmap visualization was generated using *pheatmap*. (**B**) Pairwise Pearson correlation analysis of stress transcriptomes based on log₂ fold change values of the same gene set, providing an independent quantitative measure of transcriptome similarity. Stv, starvation; Trp, tryptophan.

Hereafter, we performed a series of analyses aimed at comparing transcriptional changes across the five stress conditions. To minimize confounding effects arising from differences in serovar background, only the 874 genes conserved between *C. trachomatis* serovars D and L2 were included in these comparative analyses. We first generated a heatmap for the 667 genes that were differentially expressed in at least one condition (Fig. 1A). Hierarchical clustering revealed that tryptophan starvation and iron starvation formed the closest pair, reflecting their broadly similar effects on the bacterium due to nutrient starvation. Penicillin-treated cultures clustered next to this pair. In contrast, the IFNγ transcriptome was positioned farther away from the tryptophan-starvation transcriptome, an unexpected separation given the well-established link between IFNγ exposure and host-driven tryptophan starvation. The heat shock transcriptome remained the most distinct, forming a well-separated branch relative to the four chronic stress conditions.

To independently assess the relatedness among the five transcriptomes, we performed pairwise Pearson correlation analysis using the log₂ fold change values for the aforementioned 667 DEGs (Fig. 1B). Tryptophan starvation and iron starvation again showed the strongest correlation (*r* = 0.95). Penicillin treatment correlated strongly with iron starvation (*r* = 0.89) and tryptophan starvation (*r* = 0.84). The IFNγ transcriptome displayed more moderate correlations with these three conditions (r = 0.57–0.69), consistent with its more distant placement in the clustering analysis (Fig. 1A). Heat shock exhibited negative correlations with all other conditions (*r* = −0.14 to −0.29), reinforcing its distinct transcriptomic profile. Together, hierarchical clustering and correlation analysis reveal a clear reprogramming pattern among the five stress transcriptomes, with nutrient starvation- and penicillin-induced transcriptomes forming a closely related group, IFNγ forming a more separate branch, and heat shock representing the most divergent condition.

### Opposing effects of acute and chronic stress on the expression of protein synthesis genes and type III secretion system genes

**Fig 2:**
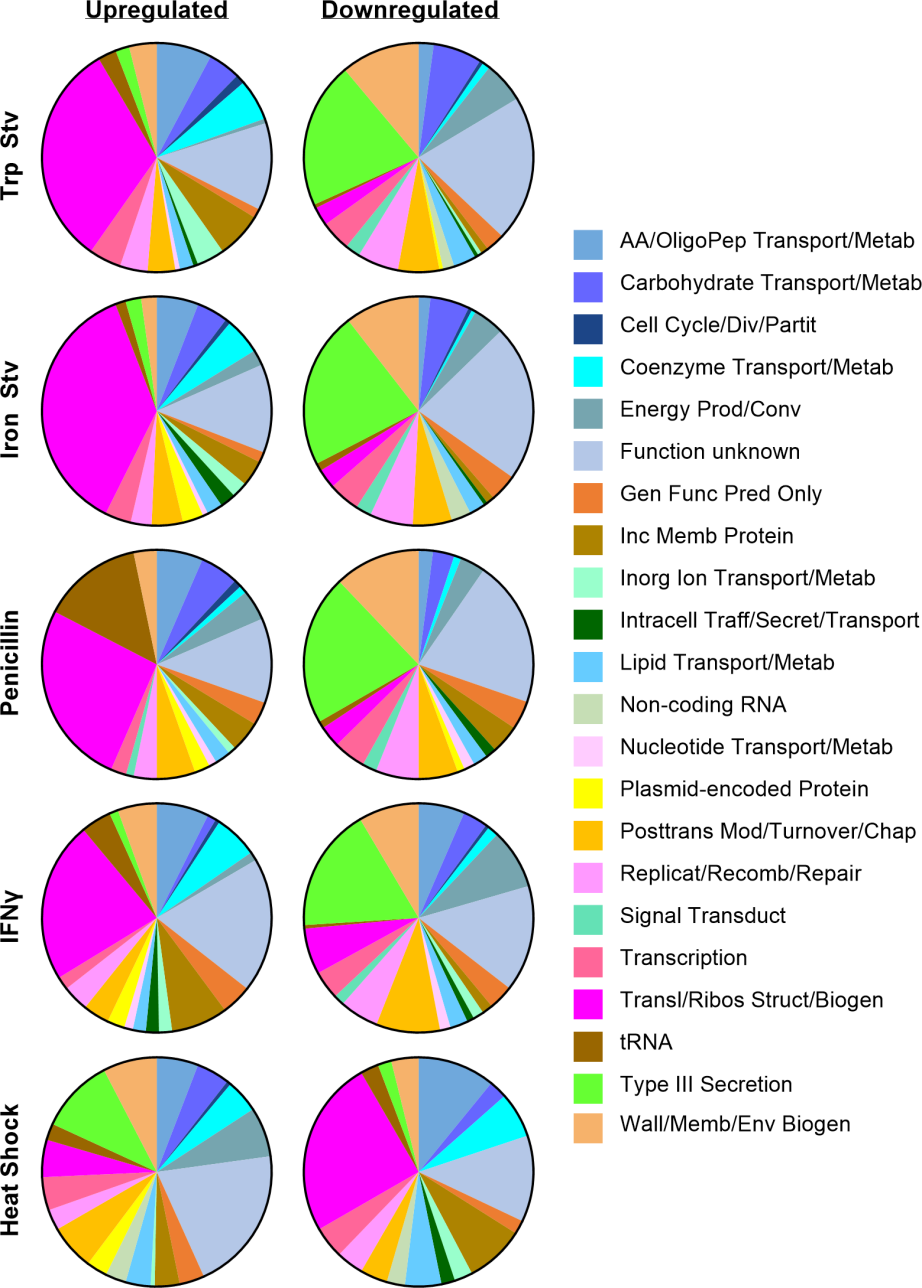
Acute and chronic stressors exert opposing effects on protein synthesis and T3SS gene expression. Pie charts show the distribution of upregulated (left) and downregulated (right) genes among gene ontology categories within each stress transcriptome. Only genes meeting differential expression criteria (adjusted *P* < 0.05; |log₂ fold change ≥ 0.585) are included. Excluding genes of unknown function, genes involved in translation and ribosomal structure and biogenesis dominate the upregulated gene sets in chronic stress transcriptomes but the downregulated gene sets in the heat shock transcriptome, whereas T3SS genes dominate the downregulated gene sets in chronic stress transcriptomes but the upregulated gene set in the heat shock transcriptome. AA, amino acid; OligoPep, oligopeptide; Metab, metabolism; Div, division; Partit, partitioning; Prod/Conv, production and conversion; Gen Func Pred, general function prediction; Inc Memb, inclusion membrane; Inorg, inorganic; Traff, trafficking; Secret, secretion; Posttrans Mod, posttranslational modification; Chap, chaperone; Replicat, replication; Recomb, recombination; Transduct, transduction; Wall/Memb/Env Biogen, wall, membrane, or envelope biogenesis.

To examine how *C. trachomatis* prioritizes biological processes under different stress conditions, we assigned the DEGs to functional categories and displayed the distributions of up- and downregulated genes as pie graphs (Fig. 2). In the IFNγ, tryptophan starvation, iron starvation, and penicillin transcriptomes, the translation and ribosomal structure and biogenesis category accounted for the largest fraction of upregulated genes, representing 23–37% of all upregulated genes under these chronic stresses. In contrast, this category contributed only 3–7% of the downregulated genes in the chronic stress transcriptomes but represented 25% of all downregulated genes under heat shock. Thus, translation and ribosomal structure and biogenesis category genes were preferentially upregulated during chronic stress but downregulated during acute heat shock.

A reciprocal pattern was observed for the type III secretion system (T3SS) category. Under the four chronic stress conditions, T3SS genes constituted only 1–3% of upregulated genes but comprised 18–22% of downregulated genes. Specifically, *copD*, *scc2*, *ctl0003/ct635*, *ctl0063/ct694*, *ctl0080/ct711*, *ctl0081/ct712*, *ctl0219/ct847*, *ctl0220/ct843*, *ctl0255/ct875*, *ctl0338/ct082*, *ctl0338A/ct083*, *ctl0397/ct142*, *ctl0398/ct143*, *ctl0883/ct619*, and *ctl0884/ct620*, which encode T3SS structural components, chaperones, or effectors, were downregulated in all four chronic stress transcriptomes (Data set S1). Previous studies have shown that these genes are upregulated during RB-to-EB differentiation (35–37). The predominant downregulation of T3SS genes during chronic stress is consistent with the persistent state, in which RB-to-EB differentiation and late developmental gene expression are suppressed. Several type III-secreted effectors participate in late developmental events linked to chlamydial exit, including Inc proteins that regulate inclusion extrusion (e.g., CT228 and MrcA) and the effector CteG, which promotes host cell lytic exit (38–42). Their downregulation under chronic stress likely helps retain persistent chlamydiae intracellularly rather than promoting their release. In contrast, heat shock at 45°C upregulated 11% of its total upregulated genes in the T3SS category while downregulating only 2% of its downregulated genes (22), suggesting that T3SS effectors normally induced during late development may contribute to intracellular chlamydial survival under this extreme stress condition.

### Stress-specific distinctions among transcriptomes

**Fig 3:**
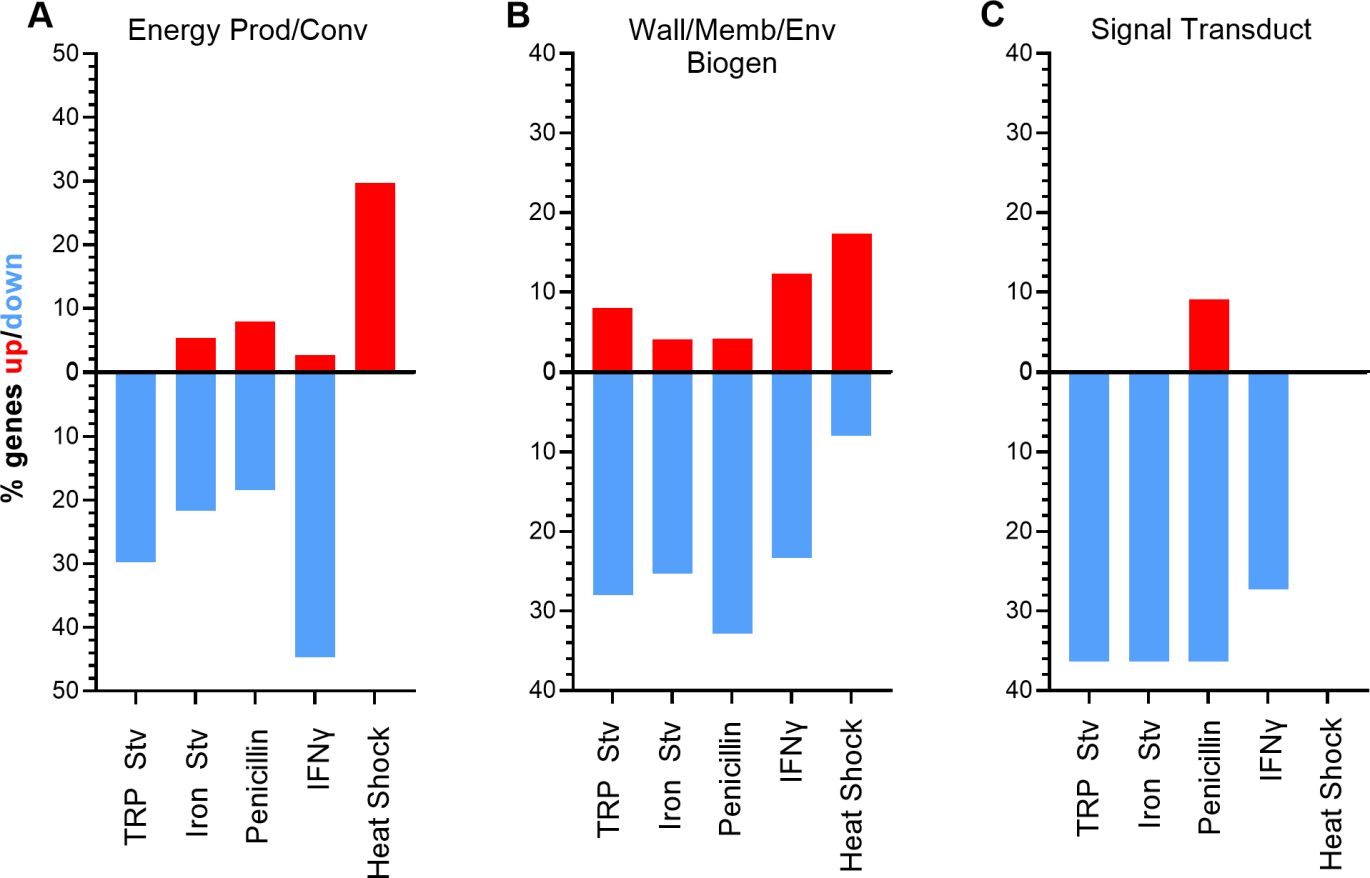
Category-normalized analysis highlights stress-specific regulation of functional gene groups. Divergent bar plots show the fraction of genes that are upregulated (red) or downregulated (blue) within selected gene ontology categories across five stress transcriptomes. Values represent the percentage of genes within each category that meet the differential expression criteria under each condition, thereby normalizing for differences in category size. Categories shown are energy production and conversion (**A**); wall, membrane, or envelope biogenesis (**B**); and signal transduction mechanisms (**C**).

To complement the DEG count-based analysis shown in the pie graphs (Fig. 2), we calculated the fraction of genes that were upregulated or downregulated within each functional category and displayed these values as divergent bar graphs (Fig. 3; Fig. S1). This category-normalized approach confirms the opposing behaviors of translation, ribosomal structure and biogenesis, and T3SS genes described above and further distinguishes the acute heat shock transcriptome from those induced by chronic stress conditions (Fig. S1). Importantly, this analysis also reveals regulatory patterns that are not apparent from comparisons based solely on absolute DEG counts.

Three functional categories displayed particularly strong differences between the chronic stress transcriptomes and the acute heat shock transcriptome, evident in both the upward and downward components of the divergent bars. Genes involved in energy production and conversion showed only small upward fractions and large downward fractions across all four chronic stress transcriptomes, whereas the heat shock transcriptome exhibited a prominent upward fraction with virtually no corresponding downregulation (Fig. 3A). This opposing pattern is consistent with reduced growth and metabolic activity under chronic stress, which likely lowers cellular energy demand, whereas survival under acute heat shock requires enhanced energy production to support stress tolerance and recovery.

Genes involved in cell wall, membrane, or envelope biogenesis exhibited consistently large downward fractions and minimal upward fractions across all four chronic stress transcriptomes, whereas the heat shock transcriptome showed a noticeable upward fraction with little corresponding downregulation (Fig. 3B). Many of the genes downregulated under chronic stress are preferentially expressed in EBs. Notably, *omcA* and *omcB*, which encode major components of the EB outer membrane complex (43), were downregulated in all four chronic stress transcriptomes but upregulated during heat shock (Data set S1). This pattern is consistent with suppression of EB-associated envelope biogenesis during chronic stress and reflects inhibition of RB-to-EB differentiation under persistence-inducing conditions. In contrast, the upregulation of *omcA* and *omcB* during acute heat shock is unexpected and may reflect a distinct stress response, possibly utilizing EB envelope components to help maintain chlamydial cellular integrity under extreme conditions.

Signal transduction mechanism genes also exhibited a clear pattern at the functional category level in the fractional analysis (Fig. 3C; Fig. S1). Across all four chronic stress conditions, this category showed a strong net downward bias, whereas heat shock induced little overall change. However, this coordinated behavior at the category level did not reflect consistent regulation of individual signaling components. For example, expression of the anti-sigma factor gene *rsbW* remained largely unchanged across stress conditions, whereas the anti-anti-sigma factor gene *rsbV2* displayed stress-dependent regulation (Data set S1). These findings suggest that signal transduction pathways are reprogrammed through selective and condition-specific regulatory adjustments across stress conditions.

Beyond this category-level trend, the fractional analysis revealed pronounced stress-specific regulation in several functional categories (Fig. S1). For example, inorganic ion transport and metabolism exhibited prominent upregulation during tryptophan starvation and, to a lesser extent, IFNγ treatment, but showed little response under iron starvation or exposure to penicillin. In contrast, tRNA genes exhibited highly variable upregulation across chronic stresses, with strong induction during penicillin treatment but minimal changes under iron starvation. These patterns underscore that, alongside common features, each stress condition elicits a distinct transcriptional response rather than a common chronic stress program.

Notably, the plasmid-encoded virulence gene *pgp3*, which encodes the secreted effector Pgp3 (44–47), and *pgp4*, which encodes a transcriptional regulator of pgp3 and numerous chromosomal genes (48, 49), exhibited stress-dependent regulation (Fig. S1). *pgp3* was downregulated during tryptophan starvation and penicillin exposure, whereas *pgp4* was repressed during penicillin treatment but induced during heat shock (Data set S1). The other plasmid genes, which primarily support plasmid maintenance, also showed stress-dependent regulation, but without a consistent pattern across conditions (Data set S1).

### Expression patterns of transcriptional regulator genes across stress transcriptomes

**Fig 4:**
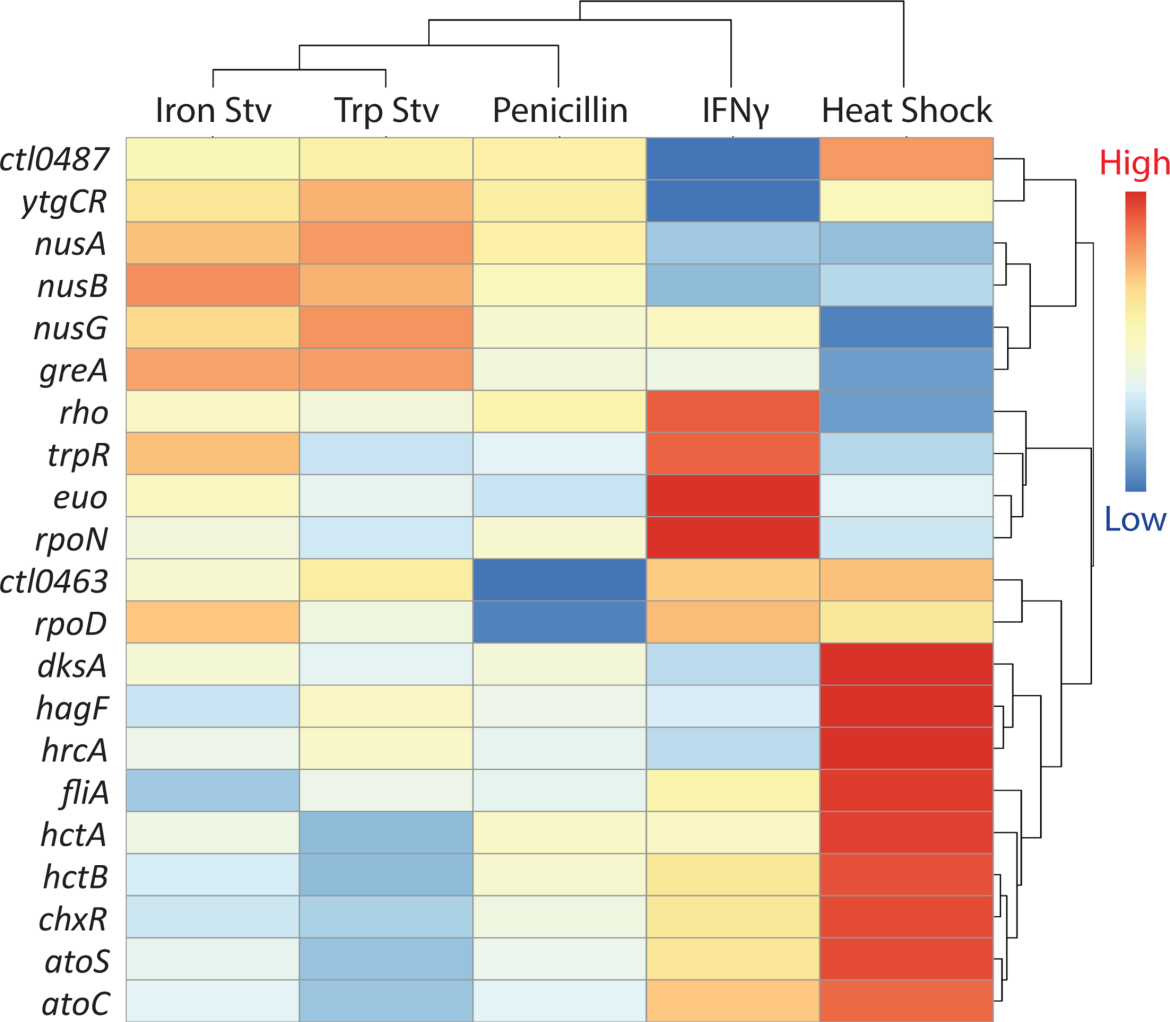
Stress-dependent expression patterns of transcriptional regulators. Hierarchical clustering heatmap of transcriptional regulator genes that are differentially expressed in at least one stress transcriptome. Clustering, dendrogram interpretation, and color scaling are as described in Fig. 1. Each row represents a transcriptional regulator, and each column represents a stress condition.

To examine how transcriptional regulation contributes to the organization of the five stress transcriptomes, we analyzed the expression patterns of transcriptional regulator genes that were differentially expressed in at least one condition and visualized their profiles by hierarchical clustering (Fig. 4). Clustering based on regulator expression closely mirrored clustering of the global transcriptomes, reinforcing the clear separation between the acute heat shock response and the four chronic stress responses.

A distinct heat shock-associated cluster comprised regulators that were strongly and selectively induced in response to heat shock. This group included *hrcA*, the heat-inducible repressor of chaperone genes (50–52), and *hagF*, a recently identified heat-responsive antagonist of *hrcA* (52). Together, these regulators define a coordinated heat shock-specific transcriptional state characterized by protein quality control.

In contrast, a chronic stress-associated cluster was defined by regulators that were preferentially elevated under subsets of chronic stress conditions relative to heat shock (Fig. 4). This group included *euo*, a repressor of late developmental gene expression (53, 54), as well as nutrient-responsive regulators, such as *trpR* and *ytgR*. Together, these patterns are consistent with sustained metabolic restriction and suppression of late developmental programs during persistence-inducing stresses (55, 56).

Beyond these broad clusters, several transcriptional regulators displayed stress-specific or heterogeneous expression patterns across chronic stress conditions (Fig. 4). The core sigma factor genes *rpoD* (σ⁶⁶), *rpoN* (σ⁵⁴), and *fliA* (σ²⁸) varied across chronic stresses rather than showing uniform regulation. Such differential regulation likely underlies features such as the partial divergence between IFNγ and tryptophan starvation transcriptomes, despite their shared impact on amino acid availability.

### Genes commonly regulated in all five stress transcriptomes

**Fig 5:**
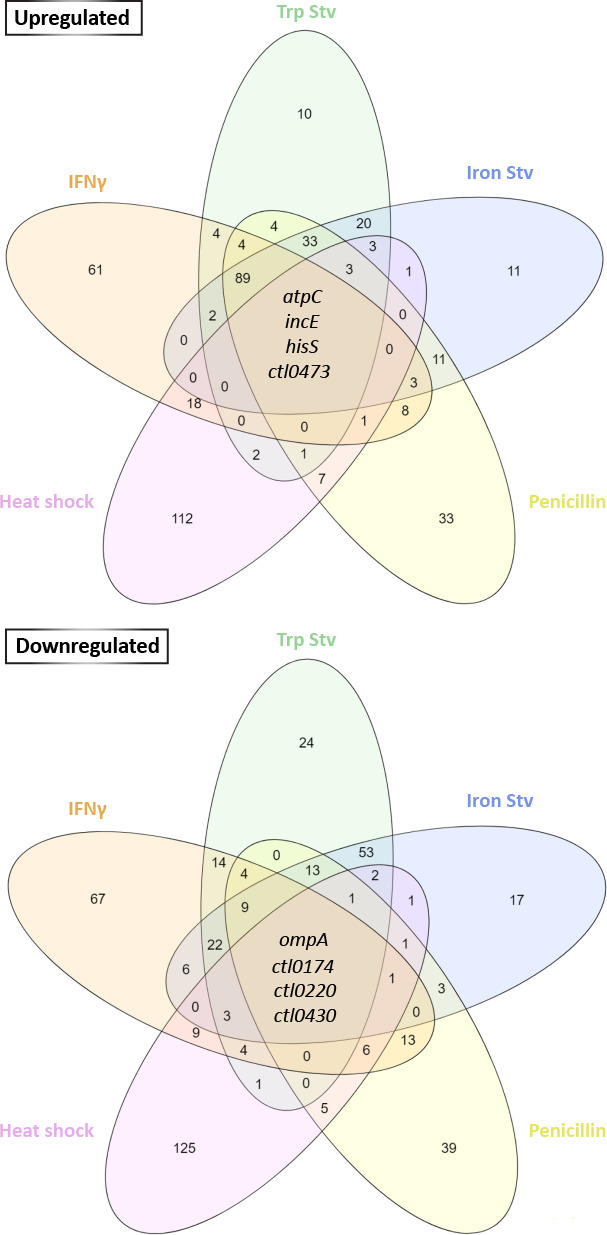
Genes commonly regulated across five stress transcriptomes. Venn diagrams show the numbers of overlapping upregulated (top) and downregulated genes (bottom) among stress transcriptomes, based on differential expression criteria applied to each transcriptome. The eight genes commonly upregulated across all five stress transcriptomes are identified.

Despite the extensive stress-specific transcriptional divergence described above, we next asked whether any genes were consistently regulated across multiple stress transcriptomes. The numbers of genes uniquely or jointly altered in the five stress transcriptomes were compared using Venn diagrams (Fig. 5). The four chronic stress transcriptomes shared 20 upregulated and 99 downregulated genes. When the heat shock transcriptome was included in the comparison, the number of commonly upregulated and commonly downregulated genes decreased to four in each group (Fig. 5).

Of the four genes commonly upregulated in all five stress transcriptomes, *atpC*, *hisS*, *incE*, and *ctl0473/ct221* encode an ATP synthase subunit, histidine-tRNA ligase, inclusion membrane protein E, and a hypothetical protein, respectively. Among the four commonly downregulated genes, *ompA* and *ctl0220/ct848* encode the major outer membrane protein (MOMP) and a T3SS effector, respectively; the remaining two genes, *ctl0174/ct805* and *ctl0430/ct178,* encode hypothetical proteins. The consistent regulation of these genes across all stress conditions suggests their involvement in stress responses, either as active drivers of adaptation or as passive markers of shared physiological states.

## DISCUSSION

Stress responses are central to chlamydial pathophysiology (12–21, 57, 58). By comparing transcriptomes derived from cultures exposed to IFNγ, tryptophan starvation, iron starvation, penicillin, or heat shock, this study provides an integrated view of how *C. trachomatis* reprograms gene expression in response to diverse adverse conditions. This comparative analysis reveals that *C. trachomatis* employs distinct transcriptional programs that reflect the nature of the biological challenge it encounters.

At a broad level, the transcriptomic relatedness among stress conditions reflects fundamental differences in the nature of the stress imposed. Consistent with this distinction, hierarchical clustering and correlation analyses reveal a clear separation between the heat shock transcriptome and the remaining stress transcriptomes. The experimental procedures used to model the heat shock response represent an acute perturbation that rapidly disrupts protein homeostasis, whereas IFNγ treatment, tryptophan starvation, iron starvation, and penicillin exposure impose prolonged limitations on growth-associated biosynthetic capacity. Accordingly, the distinct positioning of the heat shock transcriptome is not unexpected, as acute heat shock does not induce the severe developmental arrest characteristic of chlamydial persistence and was examined over a fundamentally different time course than the chronic stress conditions.

One notable feature of the chronic stress transcriptomes is the broad upregulation of ribosomal and protein synthesis genes, a pattern that contrasts with canonical bacterial stress responses (59). This behavior may be related to the absence of the *rel* gene in *Chlamydia*. In free-living bacteria, similar transcriptional profiles are typically observed only in starved *rel*-deficient mutants (2, 60, 61). Rel synthesizes the alarmone (*P*)ppGpp in response to amino acid starvation and other stressors (2, 62). The binding of (*P*)ppGpp to RNA polymerase leads to the repression of ribosomal and translational genes (2, 60, 61). Because *rel*-deficient mutants are generally more vulnerable under stress conditions (2, 60, 61), how elevated expression of ribosomal and protein synthesis genes contributes to chlamydial fitness during persistence remains an important unresolved question.

Within the group of chronic stresses, however, transcriptomic relatedness does not follow expectations based on the nominal classification of stressors, particularly the expectation that IFNγ treatment would most closely resemble tryptophan starvation. Instead, the close resemblance between tryptophan starvation and iron starvation is more pronounced than their resemblance to IFNγ treatment. This relationship can be explained by regulatory cross-talk centered on the iron-dependent transcriptional repressor YtgR (55, 56, 63). In *C. trachomatis*, translation of YtgR is gated by a tryptophan-rich regulatory motif, such that tryptophan limitation suppresses YtgR synthesis, whereas iron starvation compromises YtgR repressor activity by limiting availability of the iron cofactor required for DNA binding and repression (55, 56). As a result, both stresses converge on reduced YtgR function and a shared downstream regulatory state, providing a mechanistic basis for their close similarity.

By comparison, IFNγ treatment shows only partial overlap with tryptophan starvation. Although IFNγ-induced tryptophan depletion via host indoleamine 2,3-dioxygenase is a well-established antichlamydial mechanism, IFNγ signaling also activates additional antimicrobial programs in human cells that are not fully recapitulated by tryptophan deprivation alone, including alterations in host iron handling, metabolic reprogramming, and other cell-autonomous defenses. Supporting this, human genetic and cellular studies demonstrate that IFNγ retains substantial antimicrobial activity even when tryptophan depletion is attenuated (64). Therefore, the intermediate positioning of the IFNγ transcriptome likely reflects its multifactorial nature as a host-imposed stress that combines biosynthetic limitation with additional pressures beyond tryptophan starvation (23, 26).

Despite extensive stress-specific transcriptional reprogramming, overlap exists among transcriptomes induced by chronic stress conditions. The four chronic stress transcriptomes share approximately 120 commonly regulated genes, whereas inclusion of the heat shock transcriptome reduces this overlap to only eight genes. This graded pattern indicates that transcriptional convergence in *C. trachomatis* is stressor-dependent. Importantly, the biological significance of commonly regulated genes may differ. Consistently upregulated genes are more likely to serve functions actively involved in adaptation or survival under persistence-inducing environments, whereas commonly downregulated genes more plausibly reflect secondary consequences of growth and developmental defects.

In this context, the identification of stress-responsive pathways has implications for therapeutic intervention. Several genes that are consistently upregulated across chronic stress conditions encode functions that have been validated as antimicrobial targets in other bacterial systems. For example, bacterial tRNA synthetases are established drug targets, as illustrated by mupirocin, which inhibits isoleucyl-tRNA synthetase and is used to treat staphylococcal infections (65). Similarly, bacterial ATP synthase represents a proven vulnerability in persistent pathogens; bedaquiline targets the F₀F₁ ATP synthase of *Mycobacterium tuberculosis* and is highly effective against drug-resistant and nonreplicating bacilli (66, 67). Together, these precedents support the idea that targeting functions that are consistently upregulated and involved across multiple stress-induced transcriptional states may represent a viable strategy for disrupting chlamydial persistence.

As with any comparative analysis that integrates data sets generated under different experimental conditions, an important consideration is that the RNA-Seq studies analyzed here differed in *C. trachomatis* serovar background (D versus L2) and host cell type (human epithelial HeLa versus mouse fibroblast L929 cells), as well as in other experimental parameters inherent to the original study designs. To mitigate these sources of heterogeneity, all cross-condition comparisons presented hereafter were restricted to genes conserved between serovars D and L2 and were based on stress-induced transcriptional changes relative to matched control conditions rather than absolute expression levels. This analytical approach minimizes the impact of baseline transcriptional differences and experimental context while emphasizing biologically meaningful reprogramming associated with stress adaptation. The emergence of coherent relationships among chronic stress transcriptomes despite these differences, therefore, supports the conclusion that the observed similarities reflect shared stress-response programs rather than artifacts of study design. Nonetheless, an inherent limitation of restricting the analysis to genes shared between serovars D and L2 is that the potential contributions of serovar-specific genes to stress adaptation and persistence could not be assessed, independent of host cell background or experimental timing.

In conclusion, our findings demonstrate that adaptation to different biological stressors in *C. trachomatis* is driven by distinct transcriptomic reprogramming, while consistently involving a set of commonly regulated genes. The products of these genes may represent shared points of vulnerability across persistence-inducing environments. This work has implications for the development of future antichlamydial strategies targeting chlamydial persistence.

## MATERIALS AND METHODS

### RNA-Seq data sets and experimental conditions

Stress conditions and corresponding NCBI accession numbers for the raw RNA-Seq data sets are listed in Table 1.

### Analysis of RNA-Seq data

Raw RNA-Seq data were downloaded from the NCBI repositories listed in Table 1 and reprocessed using a unified workflow on Galaxy (68). Adapter sequences were trimmed, and low-quality reads were removed using Trimmomatic (version 0.38). Reads were aligned to either the *C. trachomatis* serovar D genome (strain UW-3/CX; chromosome: GCF_000008725.1, plasmid: NC_020986.1) or the serovar L2 genome (strain 434/Bu; chromosome: GCF_000068585.1_ASM6858v1, plasmid: AM886278) using HISAT2 (version 2.2.1). Gene expression was quantified using featureCounts (version 2.0.3) to obtain raw read counts per gene. Differential expression was assessed using DESeq2 (version 2.11.40.8) with Benjamini–Hochberg correction for multiple testing (69, 70). Genes with an adjusted *P* value (Padj) < 0.05 and a fold change ≥ 1.5 (log2FC ≥ 0.58496) were considered differentially expressed, consistent with thresholds commonly used in bacterial transcriptomic studies and in the original analyses of the data sets examined here.

### Annotation of hypothetical protein genes and their ontology assignments

Genes annotated as “hypothetical protein” in the reference genomes were evaluated to refine functional descriptions and to support ontology assignments used in the functional-category analyses. For each hypothetical protein gene, we queried ChlamBase to retrieve curated locus information, alternative gene names, and any community- or literature-derived annotations for the corresponding gene product (71). We also reviewed the corresponding UniProtKB entry to extract the current protein name, description, predicted features, and evidence context for functional annotations (72).

To incorporate experimentally supported information not captured in database summaries, we performed targeted PubMed searches using locus tags and commonly used aliases (e.g., CT_694, CTL_0360), as well as gene and protein name synonyms. When peer-reviewed studies provided direct experimental evidence (e.g., secretion via the type III secretion system, inclusion membrane localization, interaction partners, or phenotypes associated with targeted mutagenesis or knockdown), we used these findings to refine the functional description applied in this study and to guide assignment to the appropriate ontology categories (e.g., type III secretion effectors, inclusion membrane proteins, or general function prediction).

### Figure preparation

Hierarchical clustering heatmaps were generated using the R package pheatmap (73). Pearson correlation analysis was conducted in Microsoft Excel. Divergent bar graphs were created in GraphPad Prism. Venn diagrams were generated using the web-based visualization tool InteractiVenn (74).

## SUPPLEMENTAL MATERIAL

Data set S1 iai.00758-25-s0001.xlsx Supplemental data.

Fig. S1 iai.00758-25-s0002.pdf Divergent bar graphs showing the proportion of upregulated and downregulated genes within individual functional gene categories under each stress condition in *Chlamydia trachomatis*.

ASM does not own the copyrights to Supplemental Material that may be linked to, or accessed through, an article. The authors have granted ASM a non-exclusive, world-wide license to publish the Supplemental Material files. Please contact the corresponding author directly for reuse.

## References

[1] Harms A, Maisonneuve E, Gerdes K. 2016. Mechanisms of bacterial persistence during stress and antibiotic exposure. Science 354:aaf4268. doi:10.1126/science.aaf4268 27980159

[2] Das B, Bhadra RK. 2020. (p)ppGpp metabolism and antimicrobial resistance in bacterial pathogens. Front Microbiol 11:563944. doi:10.3389/fmicb.2020.563944 33162948 PMC7581866

[3] Gollan B, Grabe G, Michaux C, Helaine S. 2019. Bacterial persisters and infection: past, present, and progressing. Annu Rev Microbiol 73:359–385. doi:10.1146/annurev-micro-020518-115650 31500532

[4] Elwell C, Mirrashidi K, Engel J. 2016. Chlamydia cell biology and pathogenesis. Nat Rev Microbiol 14:385–400. doi:10.1038/nrmicro.2016.30 27108705 PMC4886739

[5] Zhong G, Brunham RC, de la Maza LM, Darville T, Deal C. 2019. National institute of allergy and infectious diseases workshop report: “Chlamydia vaccines: the way forward”. Vaccine (Auckl) 37:7346–7354. doi:10.1016/j.vaccine.2017.10.075 29097007

[6] McCullough A, Huang S, Weber MM. 2025. Pathogenicity and virulence of Chlamydia trachomatis: insights into host interactions, immune evasion, and intracellular survival. Virulence 16:2503423. doi:10.1080/21505594.2025.2503423 40353442 PMC12090877

[7] López-Hurtado M, Escarcega-Tame MA, Escobedo-Guerra MR, de Haro-Cruz MJ, Guerra-Infante FM. 2022. Identification of Chlamydia trachomatis genotypes in Mexican men with infertile women as sexual partners. Enferm Infecc Microbiol Clin (Engl Ed) 40:353–358. doi:10.1016/j.eimce.2021.02.012 35906030

[8] Bryan ER, McLachlan RI, Rombauts L, Katz DJ, Yazdani A, Bogoevski K, Chang C, Giles ML, Carey AJ, Armitage CW, Trim LK, McLaughlin EA, Beagley KW. 2019. Detection of chlamydia infection within human testicular biopsies. Hum Reprod 34:1891–1898. doi:10.1093/humrep/dez169 31586185 PMC6810529

[9] Hybiske K, Stephens RS. 2007. Mechanisms of Chlamydia trachomatis entry into nonphagocytic cells. Infect Immun 75:3925–3934. doi:10.1128/IAI.00106-07 17502389 PMC1952008

[10] Hybiske K, Stephens RS. 2007. Mechanisms of host cell exit by the intracellular bacterium Chlamydia. Proc Natl Acad Sci USA 104:11430–11435. doi:10.1073/pnas.0703218104 17592133 PMC2040915

[11] Lee JK, Enciso GA, Boassa D, Chander CN, Lou TH, Pairawan SS, Guo MC, Wan FYM, Ellisman MH, Sütterlin C, Tan M. 2018. Replication-dependent size reduction precedes differentiation in Chlamydia trachomatis. Nat Commun 9:45. doi:10.1038/s41467-017-02432-0 29298975 PMC5752669

[12] Belland RJ, Nelson DE, Virok D, Crane DD, Hogan D, Sturdevant D, Beatty WL, Caldwell HD. 2003. Transcriptome analysis of chlamydial growth during IFN-γ-mediated persistence and reactivation. Proc Natl Acad Sci USA 100:15971–15976. doi:10.1073/pnas.2535394100 14673075 PMC307677

[13] Yang C, Kari L, Lei L, Carlson JH, Ma L, Couch CE, Whitmire WM, Bock K, Moore I, Bonner C, McClarty G, Caldwell HD. 2020. Chlamydia trachomatis plasmid gene protein 3 is essential for the establishment of persistent infection and associated immunopathology. mBio 11:e01902-20. doi:10.1128/mBio.01902-20 32817110 PMC7439461

[14] Brinkworth AJ, Wildung MR, Carabeo RA. 2018. Genomewide transcriptional responses of iron-starved Chlamydia trachomatis reveal prioritization of metabolic precursor synthesis over protein translation. mSystems 3:e00184-17. doi:10.1128/mSystems.00184-17 29468197 PMC5811630

[15] Huston WM, Theodoropoulos C, Mathews SA, Timms P. 2008. Chlamydia trachomatis responds to heat shock, penicillin induced persistence, and IFN-gamma persistence by altering levels of the extracytoplasmic stress response protease HtrA. BMC Microbiol 8:190. doi:10.1186/1471-2180-8-190 18986550 PMC2585093

[16] Brockett MR, Liechti GW. 2021. Persistence alters the interaction between Chlamydia trachomatis and its host cell. Infect Immun 89:e0068520. doi:10.1128/IAI.00685-20 34001559 PMC8281235

[17] Shima K, Kaufhold I, Eder T, Käding N, Schmidt N, Ogunsulire IM, Deenen R, Köhrer K, Friedrich D, Isay SE, Grebien F, Klinger M, Richer BC, Günther UL, Deepe GS, Rattei T, Rupp J. 2021. Regulation of the mitochondrion-fatty acid axis for the metabolic reprogramming of Chlamydia trachomatis during treatment with β-lactam antimicrobials. mBio 12:e00023-21. doi:10.1128/mBio.00023-21 33785629 PMC8092193

[18] Panzetta ME, Valdivia RH, Saka HA. 2018. Chlamydia persistence: a survival strategy to evade antimicrobial effects in-vitro and in-vivo. Front Microbiol 9. doi:10.3389/fmicb.2018.03101 PMC6299033 30619180

[19] Rockey DD, Wang X, Debrine A, Grieshaber N, Grieshaber SS. 2024. Metabolic dormancy in Chlamydia trachomatis treated with different antibiotics. Infect Immun 92:e0033923. doi:10.1128/iai.00339-23 38214508 PMC10863404

[20] Kozusnik T, Adams SE, Greub G. 2024. Aberrant bodies: an alternative metabolic homeostasis allowing survivability? Microorganisms 12:495. doi:10.3390/microorganisms12030495 38543546 PMC10972484

[21] Jury B, Fleming C, Huston WM, Luu LDW. 2023. Molecular pathogenesis of Chlamydia trachomatis. Front Cell Infect Microbiol 13:1281823. doi:10.3389/fcimb.2023.1281823 37920447 PMC10619736

[22] Huang Y, Wurihan W, Lu B, Zou Y, Wang Y, Weldon K, Fondell JD, Lai Z, Wu X, Fan H. 2021. Robust heat shock response in Chlamydia lacking a typical heat shock sigma factor. Front Microbiol 12:812448. doi:10.3389/fmicb.2021.812448 35046926 PMC8762339

[23] Muramatsu MK, Brothwell JA, Stein BD, Putman TE, Rockey DD, Nelson DE. 2016. Beyond tryptophan synthase: identification of genes that contribute to Chlamydia trachomatis survival during gamma interferon-induced persistence and reactivation. Infect Immun 84:2791–2801. doi:10.1128/IAI.00356-16 27430273 PMC5038056

[24] Olaleye AO, Babah OA, Osuagwu CS, Ogunsola FT, Afolabi BB. 2020. Sexually transmitted infections in pregnancy – An update on Chlamydia trachomatis and Neisseria gonorrhoeae. Eur J Obstet Gynecol Reprod Biol 255:1–12. doi:10.1016/j.ejogrb.2020.10.002 33059307

[25] Pokorzynski ND, Alla MR, Carabeo RA. 2022. Host cell amplification of nutritional stress contributes to persistence in Chlamydia trachomatis. mBio 13:e0271922. doi:10.1128/mbio.02719-22 36377897 PMC9765610

[26] Reitano JR, Coers J. 2024. Restriction and evasion: a review of IFNγ-mediated cell-autonomous defense pathways during genital Chlamydia infection. Pathog Dis 82:ftae019. doi:10.1093/femspd/ftae019 39210512 PMC11407441

[27] Thompson CC, Carabeo RA. 2011. An optimal method of iron starvation of the obligate intracellular pathogen, Chlamydia trachomatis. Front Microbio 2. doi:10.3389/fmicb.2011.00020 PMC3109288 21687412

[28] Leslie SW, Vinod J. 2025. Lymphogranuloma venereum infection. StatPearls Publishing, Treasure Island (FL).

[29] Wu L, Chen L, Peng L, Liu C, He S, Xie L. 2025. Clinical characteristics of Chlamydia psittaci pneumonia and predictors analysis of severe patients: a retrospective observational study. Front Med 12:1565254. doi:10.3389/fmed.2025.1565254 PMC11996669 40236451

[30] Rohde G, Straube E, Essig A, Reinhold P, Sachse K. 2010. Chlamydial zoonoses. Dtsch Arztebl Int 107:174–180. doi:10.3238/arztebl.2010.0174 20358033 PMC2847324

[31] de Voux A, Kent JB, Macomber K, Krzanowski K, Jackson D, Starr T, Johnson S, Richmond D, Crane LR, Cohn J, Finch C, McFadden J, Pillay A, Chen C, Anderson L, Kersh EN. 2016. Notes from the field: cluster of lymphogranuloma venereum cases among men who have sex with men — Michigan, August 2015–April 2016. MMWR Morb Mortal Wkly Rep 65:920–921. doi:10.15585/mmwr.mm6534a6 27583686

[32] Dan M, Tyrrell LD, Goldsand G. 1987. Isolation of Chlamydia trachomatis from the liver of a patient with prolonged fever. Gut 28:1514–1516. doi:10.1136/gut.28.11.1514 3428679 PMC1433683

[33] Narberhaus F. 1999. Negative regulation of bacterial heat shock genes. Mol Microbiol 31:1–8. doi:10.1046/j.1365-2958.1999.01166.x 9987104

[34] Gragerov A, Nudler E, Komissarova N, Gaitanaris GA, Gottesman ME, Nikiforov V. 1992. Cooperation of GroEL/GroES and DnaK/DnaJ heat shock proteins in preventing protein misfolding in Escherichia coli. Proc Natl Acad Sci USA 89:10341–10344. doi:10.1073/pnas.89.21.10341 1359538 PMC50334

[35] Lu B, Wang Y, Wurihan W, Cheng A, Yeung S, Fondell JD, Lai Z, Wan D, Wu X, Li WV, Fan H. 2024. Requirement of GrgA for Chlamydia infectious progeny production, optimal growth, and efficient plasmid maintenance. mBio 15:e0203623. doi:10.1128/mbio.02036-23 38112466 PMC10790707

[36] Hatch ND, Ouellette SP. 2023. Identification of the alternative sigma factor regulons of Chlamydia trachomatis using multiplexed CRISPR interference. mSphere 8:e0039123. doi:10.1128/msphere.00391-23 37747235 PMC10597470

[37] Soules KR, LaBrie SD, May BH, Hefty PS. 2020. Sigma 54-regulated transcription is associated with membrane reorganization and type III secretion effectors during conversion to infectious forms of Chlamydia trachomatis. mBio 11:e01725-20. doi:10.1128/mBio.01725-20 32900805 PMC7482065

[38] Rucks EA. 2023. Type III secretion in Chlamydia. Microbiol Mol Biol Rev 87:e0003423. doi:10.1128/mmbr.00034-23 37358451 PMC10521360

[39] Lutter EI, Barger AC, Nair V, Hackstadt T. 2013. Chlamydia trachomatis inclusion membrane protein CT228 recruits elements of the myosin phosphatase pathway to regulate release mechanisms. Cell Rep 3:1921–1931. doi:10.1016/j.celrep.2013.04.027 23727243 PMC3700685

[40] Yang C, Starr T, Song L, Carlson JH, Sturdevant GL, Beare PA, Whitmire WM, Caldwell HD. 2015. Chlamydial lytic exit from host cells is plasmid regulated. mBio 6:e01648-15. doi:10.1128/mBio.01648-15 26556273 PMC4659467

[41] Nguyen PH, Lutter EI, Hackstadt T. 2018. Chlamydia trachomatis inclusion membrane protein MrcA interacts with the inositol 1,4,5-trisphosphate receptor type 3 (ITPR3) to regulate extrusion formation. PLoS Pathog 14:e1006911. doi:10.1371/journal.ppat.1006911 29543918 PMC5854415

[42] Pereira IS, Pais SV, Borges V, Borrego MJ, Gomes JP, Mota LJ. 2022. The type III secretion effector CteG mediates host cell lytic exit of Chlamydia trachomatis. Front Cell Infect Microbiol 12:902210. doi:10.3389/fcimb.2022.902210 35903198 PMC9318579

[43] Liu X, Afrane M, Clemmer DE, Zhong G, Nelson DE. 2010. Identification of Chlamydia trachomatis outer membrane complex proteins by differential proteomics. J Bacteriol 192:2852–2860. doi:10.1128/JB.01628-09 20348250 PMC2876478

[44] Zhang T, Huo Z, Ma J, He C, Zhong G. 2019. The plasmid-encoded pGP3 promotes Chlamydia evasion of acidic barriers in both stomach and vagina. Infect Immun 87:e00844-18. doi:10.1128/IAI.00844-18 30858342 PMC6479032

[45] Liu Y, Huang Y, Yang Z, Sun Y, Gong S, Hou S, Chen C, Li Z, Liu Q, Wu Y, Baseman J, Zhong G. 2014. Plasmid-encoded Pgp3 is a major virulence factor for Chlamydia muridarum to induce hydrosalpinx in mice. Infect Immun 82:5327–5335. doi:10.1128/IAI.02576-14 25287930 PMC4249284

[46] Zhong G. 2017. Chlamydial plasmid-dependent pathogenicity. Trends Microbiol 25:141–152. doi:10.1016/j.tim.2016.09.006 27712952 PMC5272858

[47] Turman BJ, Darville T, O’Connell CM. 2023. Plasmid-mediated virulence in Chlamydia. Front Cell Infect Microbiol 13:1251135. doi:10.3389/fcimb.2023.1251135 37662000 PMC10469868

[48] Song L, Carlson JH, Whitmire WM, Kari L, Virtaneva K, Sturdevant DE, Watkins H, Zhou B, Sturdevant GL, Porcella SF, McClarty G, Caldwell HD. 2013. Chlamydia trachomatis plasmid-encoded Pgp4 is a transcriptional regulator of virulence-associated genes. Infect Immun 81:636–644. doi:10.1128/IAI.01305-12 23319558 PMC3584862

[49] Zhang Q, Rosario CJ, Sheehan LM, Rizvi SM, Brothwell JA, He C, Tan M. 2020. The repressor function of the Chlamydia late regulator EUO is enhanced by the plasmid-encoded protein Pgp4. J Bacteriol 202:e00793-19. doi:10.1128/JB.00793-19 31988079 PMC7099128

[50] Wilson AC, Wu CC, Yates JR III, Tan M. 2005. Chlamydial GroEL autoregulates its own expression through direct interactions with the HrcA repressor protein. J Bacteriol 187:7535–7542. doi:10.1128/JB.187.21.7535-7542.2005 16237037 PMC1272993

[51] Wilson AC, Tan M. 2004. Stress response gene regulation in Chlamydia is dependent on HrcA-CIRCE interactions. J Bacteriol 186:3384–3391. doi:10.1128/JB.186.11.3384-3391.2004 15150223 PMC415772

[52] Huang Y, Wang Y, Pan M, Wan D, Wang L, Fondell JD, Wu X, Zhong G, Fan H. 2025. A lineage-specific heat-induced feedback loop controls HrcA to promote chlamydial fitness under stress. bioRxiv. doi:10.1101/2025.05.30.657042

[53] Hakiem OR, Rizvi SMA, Ramirez C, Tan M. 2023. Euo is a developmental regulator that represses late genes and activates midcycle genes in Chlamydia trachomatis. mBio 14:e0046523. doi:10.1128/mbio.00465-23 37565751 PMC10653925

[54] Rosario CJ, Hanson BR, Tan M. 2014. The transcriptional repressor EUO regulates both subsets of Chlamydia late genes. Mol Microbiol 94:888–897. doi:10.1111/mmi.12804 25250726 PMC4428689

[55] Pokorzynski ND, Brinkworth AJ, Carabeo R. 2019. A bipartite iron-dependent transcriptional regulation of the tryptophan salvage pathway in Chlamydia trachomatis. eLife 8:e42295. doi:10.7554/eLife.42295 30938288 PMC6504234

[56] Pokorzynski ND, Hatch ND, Ouellette SP, Carabeo RA. 2020. The iron-dependent repressor YtgR is a tryptophan-dependent attenuator of the trpRBA operon in Chlamydia trachomatis. Nat Commun 11:6430. doi:10.1038/s41467-020-20181-5 33353937 PMC7755916

[57] Mirrashidi KM, Elwell CA, Verschueren E, Johnson JR, Frando A, Von Dollen J, Rosenberg O, Gulbahce N, Jang G, Johnson T, Jäger S, Gopalakrishnan AM, Sherry J, Dunn JD, Olive A, Penn B, Shales M, Cox JS, Starnbach MN, Derre I, Valdivia R, Krogan NJ, Engel J. 2015. Global mapping of the Inc-human interactome reveals that retromer restricts Chlamydia infection. Cell Host Microbe 18:109–121. doi:10.1016/j.chom.2015.06.004 26118995 PMC4540348

[58] Olson-Wood MG, Jorgenson LM, Ouellette SP, Rucks EA. 2021. Inclusion membrane growth and composition are altered by overexpression of specific inclusion membrane proteins in Chlamydia trachomatis L2. Infect Immun 89:e0009421. doi:10.1128/IAI.00094-21 33875478 PMC8208519

[59] Durfee T, Hansen AM, Zhi H, Blattner FR, Jin DJ. 2008. Transcription profiling of the stringent response in Escherichia coli. J Bacteriol 190:1084–1096. doi:10.1128/JB.01092-07 18039766 PMC2223561

[60] Goodman D. 1970. Ribosomal protein synthesis during amino acid starvation and chloramphenicol treatment. J Mol Biol 51:491–499. doi:10.1016/0022-2836(70)90003-3 4923858

[61] Dalebroux ZD, Swanson MS. 2012. ppGpp: magic beyond RNA polymerase. Nat Rev Microbiol 10:203–212. doi:10.1038/nrmicro2720 22337166 PMC13198741

[62] Gratani FL, Horvatek P, Geiger T, Borisova M, Mayer C, Grin I, Wagner S, Steinchen W, Bange G, Velic A, Maček B, Wolz C. 2018. Regulation of the opposing (p)ppGpp synthetase and hydrolase activities in a bifunctional RelA/SpoT homologue from Staphylococcus aureus. PLoS Genet 14:e1007514. doi:10.1371/journal.pgen.1007514 29985927 PMC6053245

[63] Akers JC, HoDac H, Lathrop RH, Tan M. 2011. Identification and functional analysis of CT069 as a novel transcriptional regulator in Chlamydia. J Bacteriol 193:6123–6131. doi:10.1128/JB.05976-11 21908669 PMC3209234

[64] Murray HW, Szuro-Sudol A, Wellner D, Oca MJ, Granger AM, Libby DM, Rothermel CD, Rubin BY. 1989. Role of tryptophan degradation in respiratory burst-independent antimicrobial activity of gamma interferon-stimulated human macrophages. Infect Immun 57:845–849. doi:10.1128/iai.57.3.845-849.1989 2492973 PMC313187

[65] Simmons KJ, Chopra I, Fishwick CWG. 2010. Structure-based discovery of antibacterial drugs. Nat Rev Microbiol 8:501–510. doi:10.1038/nrmicro2349 20551974

[66] Melander RJ, Zurawski DV, Melander C. 2018. Narrow-spectrum antibacterial agents. Medchemcomm 9:12–21. doi:10.1039/C7MD00528H 29527285 PMC5839511

[67] Vestergaard M, Bald D, Ingmer H. 2022. Targeting the ATP synthase in bacterial and fungal pathogens: beyond Mycobacterium tuberculosis. J Glob Antimicrob Resist 29:29–41. doi:10.1016/j.jgar.2022.01.026 35131507

[68] Abueg LAL, Afgan E, Allart O, Awan AH, Bacon WA, Baker D, Bassetti M, Batut B, Bernt M, Blankenberg D, et al.. 2024. The Galaxy platform for accessible, reproducible, and collaborative data analyses: 2024 update. Nucleic Acids Res 52:W83–W94. doi:10.1093/nar/gkae410 38769056 PMC11223835

[69] Love M, Anders S, Huber W. 2014. Differential analysis of count data the DESeq2 package. Genome Biol 15:1–41. doi:10.1186/s13059-014-0550-8

[70] Love MI, Huber W, Anders S. 2014. Moderated estimation of fold change and dispersion for RNA-seq data with DESeq2. Genome Biol 15:550. doi:10.1186/s13059-014-0550-8 25516281 PMC4302049

[71] Putman T, Hybiske K, Jow D, Afrasiabi C, Lelong S, Cano MA, Wu C, Su AI. 2019. ChlamBase: a curated model organism database for the Chlamydia research community. Database (Oxford) 2019:baz041. doi:10.1093/database/baz041 30985891 PMC6463448

[72] Bateman A, Martin M-J, Orchard S, Magrane M, Agivetova R, Ahmad S, Alpi E, Bowler-Barnett EH, Britto R, Bursteinas B, et al.. 2021. UniProt: the universal protein knowledgebase in 2021. Nucleic Acids Res 49:D480–D489. doi:10.1093/nar/gkaa1100 33237286 PMC7778908

[73] Kolde R. 2010. Pheatmap: pretty heatmaps. 10.32614/CRAN.package.pheatmap.

[74] Heberle H, Meirelles GV, da Silva FR, Telles GP, Minghim R. 2015. InteractiVenn: a web-based tool for the analysis of sets through Venn diagrams. BMC Bioinformatics 16:169. doi:10.1186/s12859-015-0611-3 25994840 PMC4455604

