# Supplementary material for: Comparative transcriptomics reveals genes commonly induced by distinct stressors in Chlamydia
Source: Infect Immun. 2026 Feb 20;94(3):e00758-25. doi: 10.1128/iai.00758-25 (PMC12974147; doi:10.1128/iai.00758-25)
Supplement: Fig. S1 — Divergent bar graphs showing the proportion of upregulated and downregulated genes within individual functional gene categories under each stress condition in Chlamydia trachomatis. [file iai.00758-25-s0002.pdf]

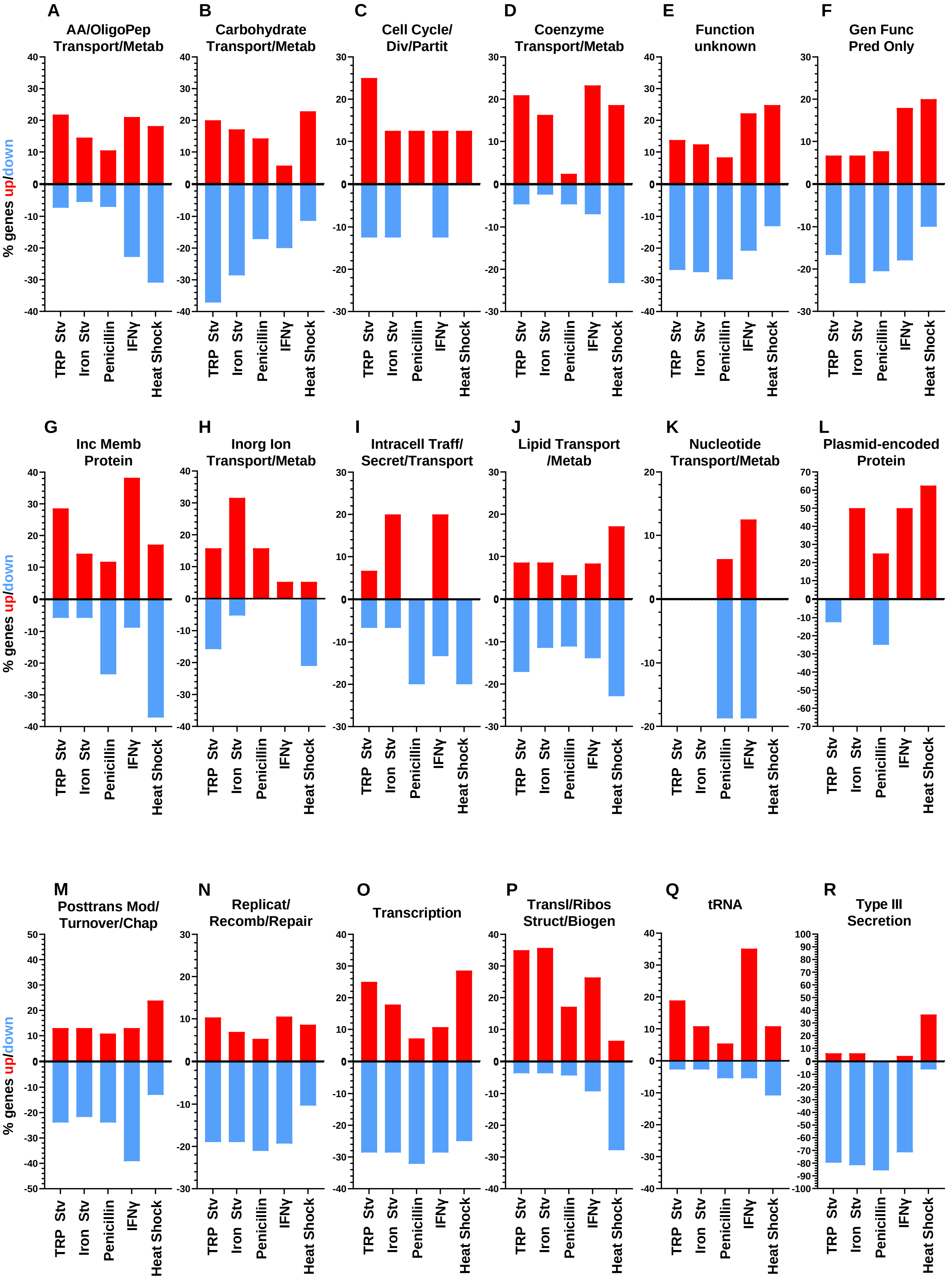
A
AA/OligoPep Transport/Metab
B
Carbohydrate Transport/Metab
C
Cell Cycle/ Div/Partit
D
Coenzyme Transport/Metab
E
Function unknown
F
Gen Func Pred Only
G
Inc Memb Protein
H
Inorg Ion Transport/Metab
I
Intracell Traff/ Secret/Transport
J
Lipid Transport /Metab
K
Nucleotide Transport/Metab
L
Plasmid-encoded Protein
M
Posttrans Mod/ Turnover/Chap
N
Replicat/ Recomb/Repair
O
Transcription
P
Transl/Ribos Struct/Biogen
Q
tRNA
R
Type III Secretion
% genes up/down
TRP Stv
Iron Stv
Penicillin
IFNγ
Heat Shock

Figure S1. Divergent bar graphs showing the proportion of upregulated and downregulated genes within individual functional gene categories under each stress condition in *Chlamydia trachomatis*.

Supplemental to Haines R, Wan D, Zhong G, Fan H. 2026. *Comparative Transcriptomics Reveals Genes Commonly Induced by Distinct Stressors in Chlamydia*. *Infect Immun*. This figure displays transcript changes in *C. trachomatis* in response to five stress conditions: tryptophan starvation, iron starvation, penicillin treatment, interferon-γ exposure, and heat shock. Each panel represents a distinct functional gene category, with red bars indicating the percentage of genes upregulated (≥1.5-fold) and blue bars indicating those downregulated (≤–1.5-fold) relative to matched controls. Categories include all functional groups except energy production and conversion, cell wall/membrane/envelope biogenesis, and signal transduction, which are presented in Figure 3 of the main manuscript. Gene sets were restricted to orthologs conserved between serovars D and L2.
